# Supplementary material for: Knowledge graph analytics platform with LINCS and IDG for Parkinson's disease target illumination
Source: BMC Bioinformatics. 2022 Jan 12;23:37. doi: 10.1186/s12859-021-04530-9 (PMC8756622; doi:10.1186/s12859-021-04530-9)
Supplement: Supplementary file 1 — Additional file 1. Top scoring 100 genes associated with PD, and drugs indicated for PD with structures and properties. [file 12859_2021_4530_MOESM1_ESM.pdf]

Additional File 1

Title: Knowledge graph analytics platform with LINCS and IDG for Parkinson's disease target illumination

Authors: Jeremy J Yang, Christopher R Gessner, Joel L Duerksen, Daniel Biber, Jessica L Binder, Murat Ozturk, Brian Foote, Robin McEntire, Kyle Stirling, Ying Ding and David J Wild

Supplementary materials **2**

- Top scoring 100 genes associated with Parkinson's disease 2
- Drugs indicated for Parkinson's disease and with ATC "NERVOUS SYSTEM" with structures and properties 6

Supplementary materials

Top scoring 100 genes associated with Parkinson's disease

Full results with all associated genes are provided as Additional File 2 (KGAP_gene_results.tsv) and Additional File 3 (KGAP_gene_results.xlsx).

	Symbol	Family	Name	KGAP Score	IDG TDL	TINX nds_rank	TINX Importance	TINX Novelty
1	MYLK	Kinase	Myosin light chain kinase, smooth muscle	50.2615	Tchem	77	0.0385	0.0013
2	CXCL2		C-X-C motif chemokine 2	48.7216	Tbio	100	0.0100	0.0030
3	SNAP25		Synaptosomal-associated protein 25	46.0216	Tclin	21	0.5410	0.0021
4	SPP1		Osteopontin	45.5870	Tbio	38	0.6174	0.0002
5	PAK1	Kinase	Serine/threonine-protein kinase PAK 1	44.6944	Tchem	80	0.0286	0.0030
6	SRC	Kinase	Proto-oncogene tyrosine-protein kinase Src	43.9768	Tclin	21	1.4295	0.0001
7	IDE	Enzyme	Insulin-degrading enzyme	42.9433	Tchem	55	0.1012	0.0025
8	ST3GAL5	Enzyme	Lactosylceramide alpha-2,3-sialyltransferase	42.2349	Tbio	40	0.0455	0.0110
9	NR3C1	NR	Glucocorticoid receptor	41.6000	Tclin	30	0.8131	0.0003
10	MAP7		Ensconsin	38.3868	Tbio	38	0.0204	0.0274
11	HBB		Hemoglobin subunit beta	37.9577	Tbio	42	0.4245	0.0004
12	BMP4		Bone morphogenetic protein 4	37.9529	Tchem	47	0.2492	0.0006
13	ADRB2	GPCR	Beta-2 adrenergic receptor	37.0831	Tclin	28	0.9421	0.0004
14	SLC1A4	Transporter	Neutral amino acid transporter A	36.8827	Tbio	44	0.0286	0.0164
15	SLC1A4	Transporter	Neutral amino acid transporter A	36.8827	Tbio	44	0.0286	0.0164
16	NOTCH1		Neurogenic locus notch homolog protein 1	36.5929	Tchem	30	0.9557	0.0001
17	MAMLD1		Mastermind-like domain-containing protein 1	36.3975	Tbio	34	0.1000	0.0095

18	GPER1	GPCR	G-protein coupled estrogen receptor 1	35.8607	Tchem	21	0.6166	0.0015
19	MCOLN1	IC	Mucolipin-1	35.2058	Tchem	58	0.0788	0.0028
20	PTGS2	Enzyme	Prostaglandin G/H synthase 2	34.9171	Tclin	10	8.6930	0.0001
21	PTGS2	Enzyme	Prostaglandin G/H synthase 2	34.9171	Tclin	10	8.6930	0.0001
22	SLC25A14	Transporter	Brain mitochondrial carrier protein 1	34.6732	Tbio	17	0.1250	0.0313
23	EPHB2	Kinase	Ephrin type-B receptor 2	34.5689	Tchem	24	0.5397	0.0019
24	SIRT3	Epigenetic	NAD-dependent protein deacetylase sirtuin-3, mitochondrial	34.2580	Tchem	16	1.1718	0.0013
25	IL1B		Interleukin-1 beta	32.9557	Tclin	5	20.7147	0.0000
26	STAP2		Signal-transducing adaptor protein 2	32.8113	Tbio	53	0.0370	0.0061
27	AGL		Glycogen debranching enzyme	31.0804	Tbio	66	0.0588	0.0024
28	NFATC3	TF	Nuclear factor of activated T-cells, cytoplasmic 3	30.8690	Tbio	36	0.1111	0.0076
29	PROS1		Vitamin K-dependent protein S	30.8634	Tbio	61	0.1052	0.0010
30	ADGRE5	GPCR	CD97 antigen	30.2677	Tbio	52	0.0159	0.0132
31	ADO	Enzyme	2-aminoethanethiol dioxygenase	30.0236	Tbio	49	0.1429	0.0019
32	CNOT4		CCR4-NOT transcription complex subunit 4	29.6965	Tbio	28	0.3537	0.0021
33	IKBKE	Kinase	Inhibitor of nuclear factor kappa-B kinase subunit epsilon	29.4758	Tchem	48	0.0139	0.0184
34	DFFB		DNA fragmentation factor subunit beta	28.8786	Tbio	71	0.0083	0.0062
35	RELB	TF	Transcription factor RelB	28.5684	Tbio	58	0.0714	0.0032
36	CDK6	Kinase	Cyclin-dependent kinase 6	28.5364	Tclin	58	0.1052	0.0017
37	TIAM1		T-lymphoma invasion and metastasis-inducing protein 1	27.6093	Tbio	59	0.0357	0.0053
38	SYNE2		Nesprin-2	27.5690	Tbio	108	0.0044	0.0019

39	MAP2K 5	Kinase	Dual specificity mitogen-activated protein kinase kinase 5	27.5558	Tchem	46	0.0385	0.0091
40	ADAM1 0	Enzyme	Disintegrin and metalloproteinase domain-containing protein 10	27.4248	Tchem	61	0.0893	0.0021
41	LYN	Kinase	Tyrosine-protein kinase Lyn	27.3761	Tclin	49	0.1447	0.0014
42	RAC2	Enzyme	Ras-related C3 botulinum toxin substrate 2	26.4949	Tbio	73	0.0635	0.0012
43	HMOX1	Enzyme	Heme oxygenase 1	26.3646	Tchem	8	9.5207	0.0002
44	HLA-DR A		HLA class II histocompatibility antigen, DR alpha chain	26.2979	Tbio	7	1.1049	0.0071
45	CASP2	Enzyme	Caspase-2	26.2581	Tchem	25	0.4140	0.0022
46	STAT3	TF	Signal transducer and activator of transcription 3	25.7421	Tchem	22	1.3099	0.0001
47	CXCR4	GPCR	C-X-C chemokine receptor type 4	25.3096	Tclin	49	0.2897	0.0003
48	CXCR4	GPCR	C-X-C chemokine receptor type 4	25.3096	Tclin	49	0.2897	0.0003
49	GNA15		Guanine nucleotide-binding protein subunit alpha-15	24.9719	Tbio	69	0.0680	0.0018
50	HP		Haptoglobin	24.7161	Tbio	39	0.5516	0.0002
51	SYNGR 3		Synaptogyrin-3	24.2133	Tdark	6	0.0524	0.2604
52	SOX2	TF	Transcription factor SOX-2	24.0188	Tbio	24	1.0835	0.0005
53	ETV1	TF	ETS translocation variant 1	23.8861	Tbio	34	0.1429	0.0070
54	MNAT1	Enzyme	CDK-activating kinase assembly factor MAT1	23.8825	Tbio	51	0.0417	0.0072
55	MNAT1	Enzyme	CDK-activating kinase assembly factor MAT1	23.8825	Tbio	51	0.0417	0.0072
56	PRKAG 2	Enzyme	5'-AMP-activated protein kinase subunit gamma-2	23.8621	Tbio	28	0.1429	0.0094
57	RRP8	Enzyme	Ribosomal RNA-processing protein 8	23.6185	Tbio	18	0.3571	0.0046
58	DAXX		Death domain-associated protein 6	23.3585	Tbio	24	0.2625	0.0043
59	CES1	Enzyme	Liver carboxylesterase 1	22.8917	Tchem	58	0.1119	0.0013

60	PIK3R3	Enzyme	Phosphatidylinositol 3-kinase regulatory subunit gamma	22.8118	Tbio	26	0.0714	0.0222
61	KIT	Kinase	Mast/stem cell growth factor receptor Kit	22.6919	Tclin	54	0.2085	0.0001
62	EGF		Pro-epidermal growth factor	22.4146	Tbio	19	1.5146	0.0002
63	LPL	Enzyme	Lipoprotein lipase	22.2644	Tchem	77	0.0623	0.0007
64	LPL	Enzyme	Lipoprotein lipase	22.2644	Tchem	77	0.0623	0.0007
65	STAT5B	TF	Signal transducer and activator of transcription 5B	21.8759	Tchem	76	0.0658	0.0009
66	GATA3	TF	Trans-acting T-cell-specific transcription factor GATA-3	21.2262	Tbio	88	0.0337	0.0009
67	CAPN1		Calpain-1 catalytic subunit	21.0522	Tchem	24	0.4176	0.0024
68	ERBB3	Kinase	Receptor tyrosine-protein kinase erbB-3	21.0286	Tclin	47	0.2109	0.0010
69	IKBKB	Kinase	Inhibitor of nuclear factor kappa-B kinase subunit beta	20.6495	Tchem	26	0.6194	0.0011
70	SYK	Kinase	Tyrosine-protein kinase SYK	20.5050	Tclin	74	0.0748	0.0007
71	NTS		Neurotensin/neuromedin N	20.1709	Tbio	10	4.1146	0.0004
72	CLPX	Enzyme	ATP-dependent Clp protease ATP-binding subunit clpX-like, mitochondrial	20.1206	Tbio	67	0.0227	0.0046
73	ACLY	Enzyme	ATP-citrate synthase	20.1079	Tclin	110	0.0058	0.0014
74	TF		Serotransferrin	20.0698	Tbio	61	0.0786	0.0023
75	LTF		Lactotransferrin	19.9048	Tbio	33	0.3492	0.0014
76	CALM3		Calmodulin-3	19.8514	Tclin	57	0.0185	0.0083
77	ALDOC	Enzyme	Fructose-bisphosphate aldolase C	19.8028	Tbio	21	0.1170	0.0214
78	CEBPA	TF	CCAAT/enhancer-binding protein alpha	19.7968	Tbio	90	0.0238	0.0010
79	SOCS2		Suppressor of cytokine signaling 2	19.3824	Tbio	61	0.0292	0.0046
80	LCN2	Enzyme	Neutrophil gelatinase-associated lipocalin	19.3678	Tbio	53	0.2036	0.0004
81	GATA2	TF	Endothelial transcription factor GATA-2	19.2315	Tbio	32	0.3113	0.0018
82	PTPRC	Enzyme	Receptor-type tyrosine-protein phosphatase C	19.0457	Tchem	28	0.9965	0.0002

83	DDR1	Kinase	Epithelial discoidin domain-containing receptor 1	18.8839	Tchem	51	0.0660	0.0048
84	EIF4G1		Eukaryotic translation initiation factor 4 gamma 1	18.6275	Tbio	7	2.4872	0.0022
85	RBP4		Retinol-binding protein 4	18.5690	Tchem	59	0.0976	0.0019
86	KYNU	Enzyme	Kynureninase	18.5648	Tchem	32	0.0373	0.0241
87	CDK5R1	Enzyme	Cyclin-dependent kinase 5 activator 1	18.5556	Tchem	21	0.1447	0.0158
88	SELL		L-selectin	18.2829	Tchem	88	0.0357	0.0004
89	PPBP		Platelet basic protein	18.0505	Tbio	91	0.0286	0.0006
90	CTTN		Src substrate cortactin	17.7222	Tbio	61	0.0847	0.0021
91	FOXO4	TF	Forkhead box protein O4	17.3937	Tbio	60	0.0476	0.0032
92	FOXO4	TF	Forkhead box protein O4	17.3937	Tbio	60	0.0476	0.0032
93	BCL2		Apoptosis regulator Bcl-2	17.2447	Tclin	24	0.2166	0.0057
94	RAB27A	Enzyme	Ras-related protein Rab-27A	17.2295	Tchem	39	0.1296	0.0046
95	CADM1		Cell adhesion molecule 1	17.2064	Tbio	109	0.0071	0.0013
96	TXNIP		Thioredoxin-interacting protein	17.1377	Tbio	33	0.2018	0.0028
97	SORBS3		Vinexin	17.0079	Tbio	28	0.0590	0.0250
98	NTRK2	Kinase	BDNF/NT-3 growth factors receptor	16.9698	Tclin	9	2.8107	0.0006
99	SERPINA1		Alpha-1-antitrypsin	16.8725	Tbio	39	0.5851	0.0002
100	PLCB3	Enzyme	1-phosphatidylinositol 4,5-bisphosphate phosphodiesterase beta-3	16.7498	Tbio	59	0.0084	0.0117

Drugs indicated for Parkinson's disease and with ATC "NERVOUS SYSTEM" with structures and properties

Also available for convenience and interoperability as Additional File 4 (PD_drugs.tsv) and Additional File 5 (PD_drugs.xlsx).

Name	DrugCent	PubChem	Molecular	SMILES	InChIKey	ATC level1	IN_LINC

	ral struct_id	CID	Depiction			class	S
amantadine	144	2130	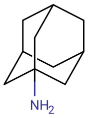	NC12CC3CC(CC(C3)C1)C2	DKNWSYNQZKUICI-UHFFFAOYSA-N	NERVOUS SYSTEM	***TRUE***
apomorphine	228	6005	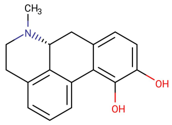	CN1CCC2=C3[C@H]1CC1=CC=C(O)C(O)=C1C3=CC=C2	VMWNQDUVQKEIOC-CYBMUJFWSA-N	NERVOUS SYSTEM	***TRUE***
benzatropine	333	1201549	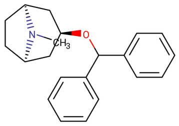	CN1[C@H]2CC[C@@H]1C[C@@H](C2)OC(C1=CC=CC=C1)C1=CC=CC=C1	GIJXKZJWITVLHI-PMOLBWCYSA-N	NERVOUS SYSTEM	***TRUE***
biperiden	374	2381	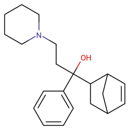	OC(CCN1CCCCC1)(C1CC2CC1C=C2)C1=CC=CC=C1	YSXKPIUOCJLQIE-UHFFFAOYSA-N	NERVOUS SYSTEM	***TRUE***
bromocriptine	403	31101	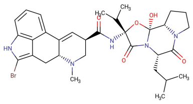	CC(C)C[C@@H]1N2C(=O)[C@](NC(=O)[C@H]3CN(C)[C@@H]4CC5=C(Br)NC6=C5C(=CC=C6)C4=C3)(O[C@@]2(O)[C@@H]2CCCN2C1=O)C(C)C	OZVBMTJYIDMWIL-AYFBDAFISA-N	NERVOUS SYSTEM	***TRUE***

dexetimide	831	30843	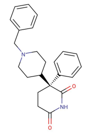	O=C1CC[C@@](C2CCN(CC3=CC=CC=C3)CC2)(C(=O)N1)C1=CC=CC=C1	LQQIVYSCPWCSSD-HSZRJFAPSA-N	NERVOUS SYSTEM	*FALSE*
entacapone	1018	5281081	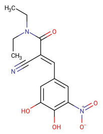	CCN(CC)C(=O)C(=C\C1=CC(=C(O)C(O)=C1)[N+]([O-])=O)\C#N	JRURYQJSLYLRLN-BJMVGYQFSA-N	NERVOUS SYSTEM	***TRUE***
levodopa	1567	6047	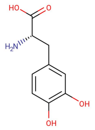	N[C@@H](CC1=CC(O)=C(O)C=C1)C(O)=O	WTDRDQBEARUVNC-LURJTMIESA-N	NERVOUS SYSTEM	***TRUE***
melevodopa	1673	23497	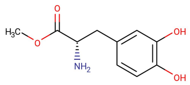	COC(=O)[C@@H](N)CC1=CC(O)=C(O)C=C1	XBBDACCLCFWBSI-ZETCQYMHSA-N	NERVOUS SYSTEM	*FALSE*
metixene	1780	4167	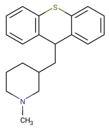	CN1CCCC(CC2C3=CC=CC=C3SC3=C2C=CC=C3)C1	MJFJKKXQDNNUJF-UHFFFAOYSA-N	NERVOUS SYSTEM	***TRUE***
opicapone	5143	135565903	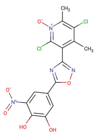	CC1=C(C2=NOC(=N2)C2=CC(O)=C(O)C(=C2)[N+]([O-])=O)C(Cl)=[N+]([O-])C(C)=C1Cl	ASOADIZOVZTJSR-UHFFFAOYSA-N	NERVOUS SYSTEM	*FALSE*
orphenadrine	1999	4601	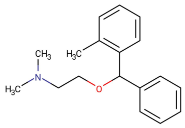	CN(C)CCOC(C1=CC=CC=C1)C1=C(C)C=CC=C1	QVYRGXJJSLMXQH-UHFFFAOYSA-N	NERVOUS SYSTEM	***TRUE***

pergolide	2105	47811	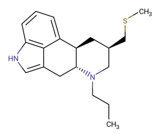	CCCN1C[C@H](CSC)C[C@H]2[C@H]1CC1=CNC3=C1C2=CC=C3	YEHCICAEULNIGD-MZMPZRCHSA-N	NERVOUS SYSTEM	***TRUE***
pimavanserin	5142	10071196	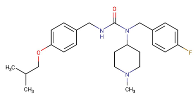	CC(C)COC1=CC=C(CNC(=O)N(CC2=CC=C(F)C=C2)C2CCN(C)CC2)C=C1	RKEWSXXUOLRFBX-UHFFFAOYSA-N	NERVOUS SYSTEM	***TRUE***
piribedil	2202	4850	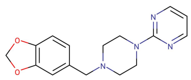	C(N1CCN(CC1)C1=NC=CC=N1)C1=CC2=C(OCO2)C=C1	OQDPVLVUJFGPGQ-UHFFFAOYSA-N	NERVOUS SYSTEM	***TRUE***
pramipexole	2233	119570	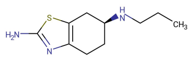	CCCN[C@H]1CCC2=C(C1)SC(N)=N2	FASDKYOPVNHBLU-ZETCQYMHSA-N	NERVOUS SYSTEM	***TRUE***
procyclidine	2276	4919	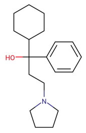	OC(CCN1CCCC1)(C1CCCCC1)C1=CC=CC=C1	WYDUSKDSKCASEF-UHFFFAOYSA-N	NERVOUS SYSTEM	***TRUE***
rasagiline	3521	3052776	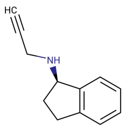	C#CCN[C@@H]1CCC2=C1C=CC=C2	RUOKEQAAGRXIBM-GFCCVEGCSA-N	NERVOUS SYSTEM	***TRUE***
rivastigmine	2392	77991	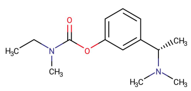	CCN(C)C(=O)OC1=CC=CC(=C1)[C@H](C)N(C)C	XSVMFMHYUFZWBK-NSHDSACASA-N	NERVOUS SYSTEM	***TRUE***
ropinirole	2402	5095	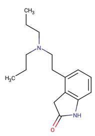	CCCN(CCC)CCC1=CC=CC2=C1CC(=O)N2	UHSKFQJFRQCDBE-UHFFFAOYSA-N	NERVOUS SYSTEM	***TRUE***

rotigotine	2407	59227	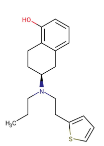	CCCN(CCC1=CC=CS1)[C@H]1CCC2=C(O)C=CC=C2C1	KFQYTPMOWPVWEJ-INIZCTEOSA-N	NERVOUS SYSTEM	***TRUE***
safinamide	4921	131682	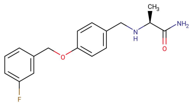	C[C@H](NCC1=CC=C(OCC2=CC(F)=CC=C2)C=C1)C(N)=O	NEMGRZFTLSKBAP-LBPRGKRZSA-N	NERVOUS SYSTEM	***TRUE***
selegiline	2429	26757	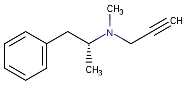	C[C@H](CC1=CC=CC=C1)N(C)CC#C	MEZLKOACVSPNER-GFCCVEGCSA-N	NERVOUS SYSTEM	***TRUE***
tolcapone	2697	4659569	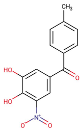	CC1=CC=C(C=C1)C(=O)C1=CC(=C(O)C(O)=C1)[N+]([O-])=O	MIQPIUSUKVNLNT-UHFFFAOYSA-N	NERVOUS SYSTEM	***TRUE***
trihexyphenidyl	2745	5572	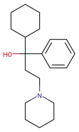	OC(CCN1CCCCC1)(C1CCCCC1)C1=CC=CC=C1	HWHLPVGTWGOCJO-UHFFFAOYSA-N	NERVOUS SYSTEM	***TRUE***
